# Predictors of response to exposure and response prevention-based cognitive behavioral therapy for obsessive-compulsive disorder

**DOI:** 10.1186/s12888-020-02841-4

**Published:** 2020-09-04

**Authors:** Sayo Hamatani, Aki Tsuchiyagaito, Masato Nihei, Yuta Hayashi, Tokiko Yoshida, Jumpei Takahashi, Sho Okawa, Honami Arai, Maki Nagaoka, Kazuki Matsumoto, Eiji Shimizu, Yoshiyuki Hirano

**Affiliations:** 1 Research Center for Child Mental Development, Chiba University, Inohana, Chuo-ku, Chiba, 2608670 Japan; 2 Japan Society for the Promotion of Science, Tokyo, Japan; 3 Laureate Institute for Brain Research, Tulsa, OK USA; 4 Department of Cognitive Behavioral Physiology, Graduate School of Medicine, Chiba University, Chiba, Japan; 5 Graduate School of Medicine and Veterinary Medicine, University of Miyazaki, Miyazaki, Japan; 6 United Graduate School of Child Development, Osaka University Research Center of Child Mental Development, Chiba University, Chiba, Japan; 7 Cognitive Behavioral Therapy Center, Chiba University Hospital, Chiba, Japan

**Keywords:** Obsessive-compulsive disorder, Exposure and response prevention, Cognitive behavioral therapy, Therapeutic response

## Abstract

**Background:**

Cognitive behavioral therapy (CBT), which includes exposure and response prevention (ERP), is effective in improving symptoms of obsessive-compulsive disorder (OCD). However, whether poor cognitive functions and autism spectrum disorder (ASD) traits affect the therapeutic response of patients with OCD to ERP-based CBT remains unclear. This study aimed to identify factors predictive of the therapeutic response of Japanese patients with OCD to ERP-based CBT.

**Methods:**

Forty-two Japanese outpatients with OCD were assessed using the Wechsler Adult Intelligence Scale-III (WAIS-III), Yale-Brown Obsessive-Compulsive Scale, Patient Health Questionnaire 9-item scale, and Autism Spectrum Quotient (AQ) at pre- and post-treatment. We used multiple regression analyses to estimate the effect on therapeutic response change. The treatment response change was set as a dependent variable in multiple regression analyses.

**Results:**

Multiple regression analyses showed that among independent variables, communication as an AQ sub-scale and Letter Number Sequencing as a WAIS-III sub-test predict the therapeutic response to ERP-based CBT .

**Conclusions:**

Our results suggest that diminished working memory (Letter Number Sequencing), poor communication skill (AQ sub-scale) may undermine responsiveness to ERP-based CBT among patients with OCD.

**Trial registration:**

UMIN, UMIN00024087. Registered 20 September 2016 - Retrospectively registered (including retrospective data).

## Background

Obsessive-compulsive disorder (OCD) is a psychiatric disorder characterized by repeated compulsive and obsessive behavior, and its 12-month prevalence in the world is 1.1 to 1.8% (DSM-5) [1]. NICE guidelines recommend the use of CBT including exposure response prevention (ERP) as a first-line of treatment for OCD, and selective serotonin reuptake inhibitors (SSRI) or more intensive CBT including ERP or combined treatment (CBT including ERP plus SSRI) for moderate to severe OCD [2]. With a treatment response change of approximately 45 to 70% [2, 3], the efficacy of the CBT including ERP has been demonstrated [4–7]. However, about 20% of OCD don’t have good enough response to ERP [3]. Numerous studies have been conducted on cognitive functions of individuals to account for their lack of response to CBT including ERP [8–11]. Neuropsychological functioning has so far been studied as a predictor of the responsiveness of patients with OCD to CBT including ERP, but the results are inconsistent [8–11]. Predictor variables of CBT including ERP for OCD can be classified into various categories [12]: demographic variables; OCD symptom characteristics such as severity; comorbidities and associated symptom severity; cognitive influences; motivational factors such as treatment expectations; treatment factors such as compliance and therapeutic alliance; biological factors; other factors such as personality, family dysfunction, and treatment-specific characteristic [12, 13].

Previous studies have suggested that responses to CBT including ERP are diminished among patients whose symptoms overlap with autism spectrum disorder (ASD) criteria [14, 15]; treatment resistance may thus be attributable the presentation of ASD characteristics. Moreover, severe major depressive disorder has been shown to inhibit therapeutic response to CBT including ERP [12]. It has also been suggested that the severity of obsessive-compulsive symptoms and beliefs may influence the response to CBT including ERP treatment [16]. Conversely, several previous studies have reported that comorbidities such as depression and anxiety do not affect treatment responsiveness to CBT including ERP [17–19]. Therefore, the results are inconsistent [12–19], and further research is needed to identify predictors of response to CBT including ERP.

Furthermore, no studies have examined the factors that affect treatment effects including the full-version of the WAIS for patients with OCD. Specifying people that need an adapted treatment strategy is very important, and it is necessary to specify predictors of treatment response. Here, the present study aimed to elucidate factors related to therapeutic responses to ERP-based CBT, focusing on ASD propensity, cognitive function, OCD severity, and depression severity.

## Methods

### Study design

**Fig. 1:**
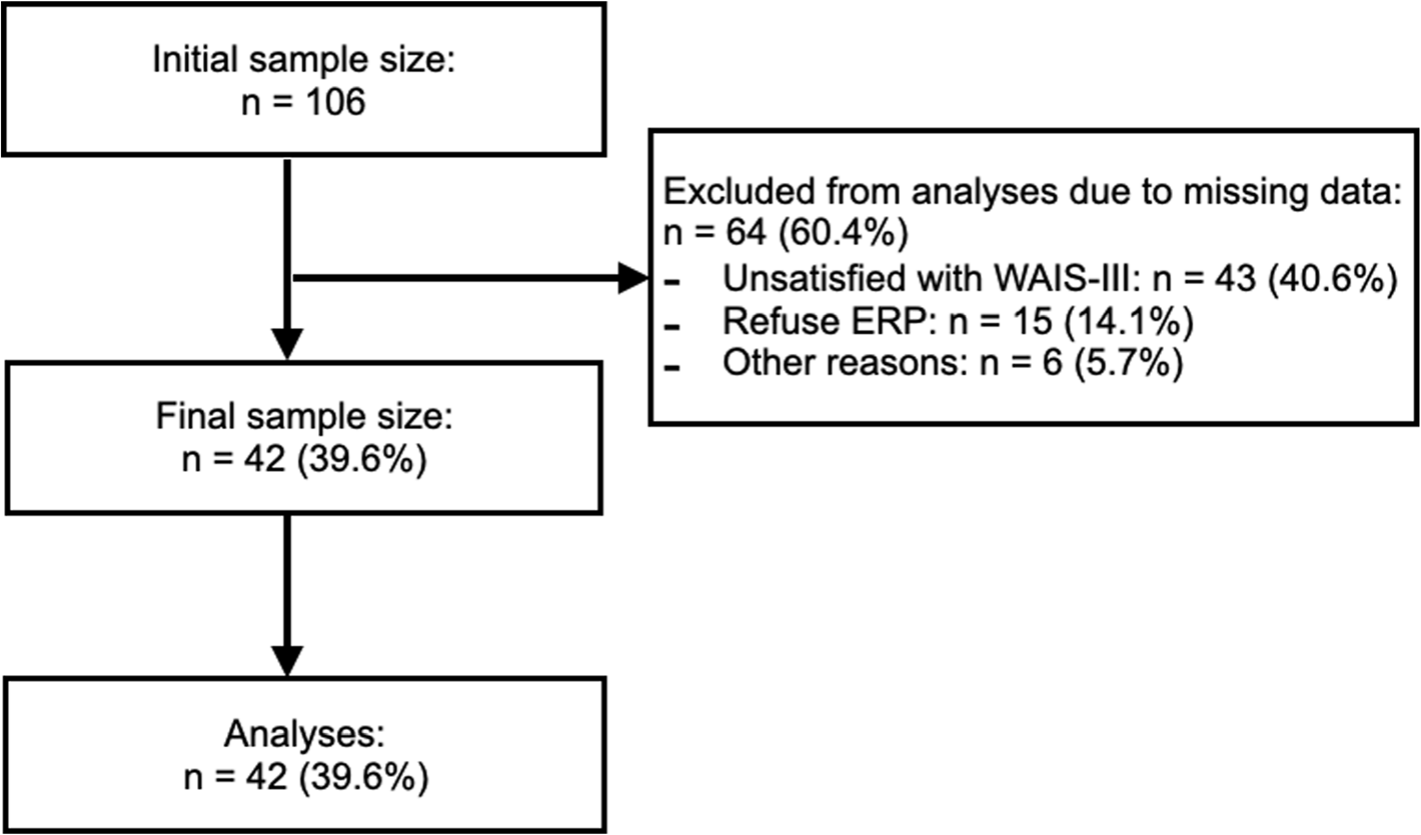
Patient flow

The present study was included patients who visited the Cognitive Behavioral Therapy Center of Chiba University between March 2013 to May 2018; it included 106 patients who were diagnosed with OCD by a psychiatrist using the Structured Clinical Interview for DSM-IV Axis I Disorders [20]. At the time of the visit to our center, the patient was already diagnosed with OCD at another institution, and he/she brought a referral letter. The diagnosis and evaluation were performed by a well-educated psychiatrist and clinical psychologist at the IAPT of Chiba University. The exclusion criteria were any organic central nervous system disorder, psychosis, intellectual disability, high risk of suicide, substance abuse or dependence, or unstable medical condition; patients for whom cognitive function could not be measured in terms of outcomes and those who did not complete the ERP intervention were also excluded. A total of 64 patients were therefore excluded, so that eventually 42 patients (mean age = 33.2 years, standard deviation =7.6 years, female = 26, male = 16) with OCD were included in the analysis (Fig. 1). Moreover, none of the participants were diagnosed with attention deficit hyperactivity disorder. Nine patients were pharmacotherapy-free, and 33 patients were taking psychotropic drugs at the time of assessment [29 patients (SSRI), 2 patients (Noradrenergic and specific serotonergic antidepressant), 5 patients (Tricyclic antidepressant), 17 patients (Benzodiazepine), 6 patients (Dopamine system stabilizer), 2 patients (Dopamine serotonin antagonist), 1 patient (Serotonin-dopamine antagonist), 3 patients (Multi-acting receptor targeted antipsychotic), 2 patients (Benzamide antipsychotics), 2 patients (Branched fatty acid), and 1 patient (Butyrophenone)](See supplemental material).

### Intervention

ERP-based CBT was performed on patients with OCD according to a treatment manual created by our research group designed for adult outpatients with OCD(https://www.mhlw.go.jp/file/06-Seisakujouhou-12200000-Shakaiengokyokushougaihokenfukushibu/0000113840.pdf). The modules were derived from a previous study on in-person ERP for OCD in Japan [7]; these modules included psychoeducation, exposure exercises, and homework assignments [7]. Sixteen ERP-based CBT sessions of 50 min in length were scheduled each week. All therapists who participated in this study completed the Improving Access to Psychological Therapies project at Chiba University [21]. The quality of ERP-based CBT was controlled through weekly group supervisions led by a psychiatrist. It was recommended that the therapist should record the content of the session using videography and an integrated chip (IC) recorder. However, it was possible for the patient to refuse to consent to this recording.

### Outcomes

#### Yale-Brown obsessive-compulsive scale

To assess the severity of the obsessive-compulsive symptoms, we used the Yale-Brown Obsessive-Compulsive Scale (Y-BOCS) [22, 23]. This scale consists of 10 items (5 obsessions and 5 compulsive items). The questionnaire items are scored on a 4-point Likert-scale; with 0 = no symptoms to 4 = extreme symptoms. The total score range is 0–40, with individual subtotals for obsessions and severity of obsessions. This scale was used in a semi-structured interview setting.

#### Obsessive-compulsive inventory

The Obsessive-Compulsive Inventory (OCI) consists of 42 items and is a 5-point Likert-scale [24, 25]. It consists of seven subscales (washing, checking, doubting, ordering, obsessions, hoarding, and neutralizing).

#### Patient health Questionnaire-9

The presence and severity of symptoms of depression experienced in the previous 2 weeks were evaluated using the Patient Health Questionnaire-9 (PHQ-9) [26, 27]. The self-administered questionnaire items are scored on a 4-point Likert-scale; with 0 = not at all to 3 = almost every day. The total score range is 0–27 (0 to 4 indicates no symptoms, 5 to 9 indicates mild symptoms, 10 to 14 indicates moderate symptoms, 15 to 19 indicates moderate to severe symptoms, and 20 to 27 indicates severe symptoms). The cut-off score for clinically significant symptoms of depression is 10.

#### Generalized anxiety disorder − 7 (GAD-7)

The presence and severity of generalized anxiety disorder was assessed using the GAD-7 [28, 29], a self-administered questionnaire that assesses the severity of generalized anxiety disorder in the previous 2 weeks on a 4-point Likert scale; with 0 = not at all to 3 = almost every day. The total score range is 0–21 (0 to 4 indicates no symptoms, 5 to 9 indicates mild symptoms, 10 to 14 indicates moderate symptoms, and 15 to 21 indicates severe symptoms). The cut-off score for clinically significant symptoms of anxiety is 10.

#### Autism-spectrum quotient

Autism-spectrum Quotient (AQ) is a self-managed instrument that can use any of the dichotomous evaluations to measure autistic characteristics [30, 31]. The total score range is 0–50. It consists of five subscales (social skills, attention switching, attention to detail, communication, and imagination). The cut-off score for clinically significant symptoms of ASD is 33.

#### Wechsler adult intelligence scale-third edition

The Wechsler Adult Intelligence Scale-third edition (WAIS-III) is a comprehensive test of intellectual functioning [32, 33]. A total of 13 subtests assessing either verbal IQ (VIQ) or performance IQ (PIQ) were administered to patients with OCD. The subtests evaluating VIQ included Vocabulary, Similarities, Information, Comprehension, Arithmetic, Digit Span, and Letter-Number Sequencing; those assessing PIQ included Picture Completion, Block Design, Matrix Reasoning, Visual Puzzles, Digit Symbol Coding, and Symbol Search. The Object Assembly subtest was excluded from the present analysis because it has a lower confidence factor than the other subtests [34]. The aforementioned subtests were grouped into the following four indices: VCI (Vocabulary, Similarities, and Information), POI (Picture Completion, Block Design, Matrix Reasoning), WMI (Digit Span and Arithmetic, and Letter-Number Sequencing), and PSI (Symbol Search and Digit Symbol Coding).

### Statistical analysis

The statistical analysis was performed using SPSS Statistics, version 26.00 (IBM Corp., Armonk, NY, USA). To investigate the predictive effects that patient pretreatment background may have had on the treatment response change post treatment, a series of analyses were performed. First, the treatment response change was obtained in terms of the difference between pre- and post-treatment Y-BOCS scores. Next, Pearson correlation coefficients were used to investigate the factors affecting the ERP-based CBT response change and to explore the relationships between such changes and other clinical variables including age, sex, severity of obsessive-compulsive symptoms in Y-BOCS at pretreatment, the traits associated with the autistic spectrum in AQ total scores or its sub-scales, intelligence index in WAIS-III or its sub-tests, OCI total score or its sub-scales, and severity of depression in PHQ-9. Finally, forward stepwise regression analysis was performed with the variables that remained significant in the correlation analysis as independent variables and the ERP-based CBT response change as the dependent variable. Moreover, the unpaired t-test was used to compare the ERP-based CBT plus pharmacotherapy group and ERP-based CBT without pharmacotherapy group, to investigate the effects of medication.

## Results

Demographic and clinical characteristics and WAIS scores of patients with OCD are shown in Table 1. The correlations between the ERP-based CBT response change and other clinical variables in OCD group are presented in Table 2. Significant differences in the ERP-based CBT response change were observed according to sex (*p* = 0.017), Attention switching (*p* = 0.029), Communication (*p* = 0.026), and Letter Number Sequencing (*p* = 0.005). No significant correlation was found between the ERP-based CBT response change and any other clinical variable. Multiple regression analysis was performed with sex, communication, attention switching, and Letter Number Sequencing as explanatory variables and the ERP-based CBT response change as the dependent variable. Multiple regression analyses showed that communication as an AQ sub-scale and Letter Number Sequencing as a WAIS-III sub-test were significant predictors of ERP-based CBT response, if sex and attention switching were excluded for a better fit (Table 3). To investigate the effects of medication, the comparison of the treatment response of the ERP-based CBT plus pharmacotherapy group and ERP-based CBT without pharmacotherapy group did not reveal any significant differences (t (40) =0.876, *p* < 0.386).

**Table 1:** Characteristics and WAIS scores in patients with OCD

	OCD	*N*
	Mean ± SD	
No. (male/female)	42 (16/26)	
Age ^b^	33.19 ± 7.55	42
Yale-Brown Obsessive-Compulsive Scale (Y-BOCS) (pre) Total	26.26 ± 4.10	42
Yale-Brown Obsessive-Compulsive Scale (Y-BOCS) (post) Total	16.00 ± 8.18	42
ERP-based CBT response change	10.26 ± 7.86	42
Obsessive Compulsive Inventory (OCI)		
*Washing*	18.59 ± 10.81	39
*Checking*	16.62 ± 9.14	39
*Doubting*	7.10 ± 4.08	39
*Ordering*	6.85 ± 4.62	39
*Obsessions*	14.26 ± 5.61	39
*Hoarding*	3.36 ± 3.17	39
*Neutralizing*	7.62 ± 5.01	39
Total	74.30 ± 26.18	40
Patient Health Questionnaire-9 (PHQ-9)	12.20 ± 5.83	41
Generalized Anxiety Disorder −7 (GAD-7)	11.93 ± 4.51	40
Autism Spectrum Quotient (AQ) AQ		
*Social skill*	5.13 ± 2.60	40
*Attention switching*	6.20 ± 2.04	40
*Attention to detail*	5.32 ± 1.82	40
*Communication*	3.95 ± 2.67	40
*Imagination*	4.08 ± 2.38	40
Total	24.68 ± 7.76	40
Wechsler Adult Intelligence Scale-III		
*Full-scale intelligence quotient (FSIQ)*	100.95 ± 10.90	42
*Verbal IQ*	102.43 ± 11.51	42
*Performance IQ*	98.88 ± 11.40	42
Indices		
Verbal Comprehension Index *(VCI)*	100.95 ± 11.77	42
Perceptual Organization Index *(POI)*	100.45 ± 12.86	42
Working Memory Index *(WMI)*	98.26 ± 16.30	42
Processing Speed Index *(PSI)*	91.17 ± 17.13	42
Subtests		
*Vocabulary*	10.52 ± 2.44	42
*Similarities*	10.55 ± 2.47	42
*Information*	9.38 ± 2.47	42
*Comprehension*	12.10 ± 2.99	42
*Arithmetic*	9.69 ± 2.67	42
*Digit Span*	10.99 ± 3.06	42
*Letter Number Sequencing*	9.86 ± 3.43	42
*Visual Puzzles*	10.48 ± 2.80	42
*Picture Completion*	9.67 ± 2.81	42
*Block Design*	9.67 ± 3.21	42
*Matrix Reasoning*	11.12 ± 2.60	42
*Digit Symbol Coding*	8.69 ± 2.97	42
*Symbol Search*	9.05 ± 2.62	42

**Table 2:** Correlations between ERP-based CBT response change and other clinical indices in OCD

	*N*	*r*	*p*-value
Age	42	0.12	0.455
Sex^a^	42	0.37^*^	0.017
Autism-Spectrum Questionnaire (AQ)			
*Social skill*	40	−0.08	0.621
*Attention switching*	40	−0.35^*^	0.029
*Attention to detail*	40	0.07	0.674
*Communication*	40	−0.35^*^	0.026
*Imagination*	40	−0.09	0.600
Total	40	−0.25	0.120
Y-BOCS (pre) Total	42	0.18	0.249
Obsessive Compulsive Inventory (OCI)			
*Washing*	39	0.18	0.264
*Checking*	39	−0.23	0.161
*Doubting*	39	−0.17	0.295
*Ordering*	39	0.00	0.994
*Obsessions*	39	−0.10	0.529
*Hoarding*	39	−0.22	0.186
*Neutralizing*	39	−0.06	0.726
Total	40	−0.09	0.562
PHQ-9	41	−0.23-0.026	0.142
GAD-7	40	−0.188	0.246
Full-scale intelligence quotient (FSIQ)	42	0.08	0.621
WAIS-III Subtests			
*Vocabulary*	42	0.17	0.269
*Similarities*	42	0.00	0.981
*Information*	42	−0.01	0.955
*Comprehension*	42	0.05	0.740
*Arithmetic*	42	0.13	0.431
*Digit Span*	42	0.07	0.699
*Letter Number Sequencing*	42	0.42^**^	0.005
*Visual Puzzles*	42	−0.14	0.365
*Picture Completion*	42	−0.10	0.539
*Block Design*	42	0.15	0.333
*Matrix Reasoning*	42	−0.13	0.418
*Digit Symbol Coding*	42	0.21	0.178
*Symbol Search*	42	0.04	0.792

*p < 0.01, **p < 0.05

*Abbreviations*: *OCD* obsessive-compulsive disorder, *Y-BOCS* Yale-Brown Obsessive-Compulsive Scale, *PHQ-9* Patient Health Questionnaire-9, *GAD-7* Generalized Anxiety Disorder-7

^a^*Female = 1. Male = 0*

**Table 3:** Results of stepwise regression analyses on response to ERP-based CBT

Dependent variable	Independent variable	Adjusted R^2^	*β*	*p*-value
Response	Communication	0.33	−0.44**	0.002
	Letter Number Sequencing		0.50**	0.001

**p < 0.01

## Discussion

The present study investigated whether clinical symptoms and cognitive functions are predictive of differential therapeutic response to ERP-based CBT among patients with OCD. We found that the ERP-based CBT response change was affected by diminished working memory as a Letter Number Sequencing and poor communication skill as an AQ subscale in Japanese participants with OCD.

A retrospective study of randomized control trials assessing 108 obsessive-compulsive patients receiving selective serotonin reuptake inhibitors reported that co-morbidity affected treatment response [35]. Our results were not consistent with those of a previous study [35]. The results of the present study suggest that depressive mood severity was excluded, but that partial ASD propensity impairs treatment response. A previous review has suggested that CBT including ERP for obsessive-compulsive disorder with ASD is effective [36], but that the response to CBT including ERP is relatively poor [15]. The novelty of this study was that the ability to communicate in AQ predicted treatment response. Without good communication, it is difficult to set appropriate therapeutic goals and exposure tasks. Therefore, it is natural that communication disorder, one of the core disorders in ASD [1], impairs treatment response.

The results of this study did not suggest that OCI’s sub-tests predict of response to ERP-based CBT. A subtype of obsessive-compulsive disorder, the hoarding state, was reported to reduce patient outcomes due to adherence [37]. Additionally, a previous study showed that reductions in obsessive beliefs influenced improvements in patients with OCD [38], which are inconsistent with the results of the present study. Previous studies suggested that patient consensus on therapeutic goals and tasks is probably also an important factor in implementing CBT including ERP [39, 40]. The present study did not measure patients’ adherence to ERP-based CBT or the degree of agreement on treatment. Future research should consider these as well. A previous representative study suggested that maleness was predictive of better treatment outcomes [41]. However, our results show that sex was not a predictor of the response to ERP-based CBT, and are consistent with some previous studies for children to adults [18, 42–44].

Although some authors have questioned whether Letter-Number Sequencing can accurately measure working memory [45], the results of the present study suggested that a subtest of working memory, “Letter Number Sequencing,” predicts treatment response. This suggests that the executive function, including working memory, of obsessive-compulsive patients undergoing ERP-based CBT may predict responsiveness. When patients with OCD have poor executive function, they might cannot understand their problem or conduct and complete ERP tasks appropriately. A previous brain imaging study showed that abnormalities in the left dorsolateral prefrontal cortex, a region that has been implicated in working memory [46], negatively affect CBT including ERP outcomes [15]. Mental flexibility, as measured using the California Verbal Learning Test, was predictive of a good response to CBT including ERP; in contrast, it was interesting to note that fluoxetine responsiveness was impaired [9]. Executive function weakness is also known to affect treatment response [9, 47–49]. The present study, for the first time in the world, has found that a WAIS-III full-version subtest, Letter Number Sequencing, predicts the response of ERP-based CBT treatment in patients with OCD. In other words, supplementing poor working memory may be beneficial for treatment and results of this study may be helpful to clinicians and cognitive behavioral practitioners choose more effective treatment strategy. In one example, to promote better responsiveness among patients with poor working memory, clinicians can provide more sessions and use visual aids during interventions [50]. Letter Number Sequencing is a simple test that can be performed in about 5 to 10 min. Therefore, clinicians and cognitive behavioral practitioners may be able to estimate response to treatment based on the results of WAIS-III Letter Number Sequencing and AQ communication score before conducting ERP-based CBT in patients with OCD.

This study had several limitations. First, while our findings implicate ASD traits as a risk factor affecting the treatment response change, cohort studies for children and early adolescents have shown that OCD is predicted by beliefs such as intolerance to uncertainty [51]. Since patients with ASD are characterized by intolerance to uncertainty, it remains unclear whether ASD traits itself is a risk factor or whether the intolerance to uncertainty accounts for the lower responsiveness to ERP-based CBT. To clarify this point, it will be necessary to also use the Obsessive Belief Questionnaire in future investigations. Second, in this study, we found that the response of ERP-based CBT was not good when the AQ communication score was high. However, AQ is a self-administered scale, and it is unclear whether this accurately reflects the communication ability. Therefore, it is necessary to measure the quality of communication objectively by behavioral observation, and not by using a self-reported scale. In the future, a more detailed assessment, including the Second Edition of the Autism Diagnosis and Observation Schedule, will be needed to identify ASD [52]. Third, the effects of the participants’ medication were not included, because their administration might have changed according to their condition during ERP-based CBT, though we asked the physicians to maintain the medication content and dose constant as much as possible. Research that regulates the content of pharmacotherapy should be conducted in the future.

Fourth, Y-BOCS evaluations were conducted by therapists who were in charge of the patients. Therefore, independent assessors would be needed to evaluate the primary outcomes, including Y-BOCS. Finally, we did not include patients who did not consent to ERP-based CBT in this study, because we could not obtain their post-treatment score (if they did not receive ERP-based CBT) or the reason for refusal.

## Conclusions

Our results suggest that diminished working memory (Letter Number Sequencing), and poor communication skill (AQ sub-scale) score may undermine responsiveness to ERP-based CBT among patients with OCD. The corresponding predictors (working memory, communication skill) of response to ERP-based CBT explain 33% of the responsiveness to ERP-based CBT among patients with OCD. To validate our findings and overcome the limitations of this study, future research should also consider the intolerance to uncertainty and the quality of ERP-based CBT.

## Supplementary information

**Additional file 1.** Pharmacotherapeutic agents used by the participants.

## Data Availability

The datasets generated and/or analysed during the current study are available in the [OSF] repository, [https://osf.io/m7hxb/].

## Abbreviations

OCD: Obsessive Compulsive Disorder
CBT: Cognitive Behavioral Therapy
ERP: Exposure and Response Prevention
WAIS-III: Wechsler Adult Intelligence Scale-III
ASD: Autism Spectrum Disorder
Y-BOCS: Yale-Brown Obsessive-Compulsive Scale
PHQ-9: Patient Health Questionnaire-9
AQ: Autism-Spectrum Questionnaire
