# Supplementary material for: Predictors of response to exposure and response prevention-based cognitive behavioral therapy for obsessive-compulsive disorder
Source: BMC Psychiatry. 2020 Sep 4;20:433. doi: 10.1186/s12888-020-02841-4 (PMC7473813; doi:10.1186/s12888-020-02841-4)
Supplement: Supplementary file 1 — Additional file 1. Pharmacotherapeutic agents used by the participants. [file 12888_2020_2841_MOESM1_ESM.docx]

Pharmacotherapeutic agents used by the participants

| **Class/type of drug** | **Specific drug** | **N** |
| --- | --- | --- |
| **SSRI** |  |  |
|  | Paroxetine hydrochloride hydrate | 12 |
|  | Sertraline hydrochloride | 7 |
|  | Fluvoxamine maleate | 9 |
|  | Escitalopram oxalate | 1 |
| **NaSSA** |  |  |
|  | Mirtazapine | 2 |
| **Tricyclic antidepressant** |  |  |
|  | Imipramine hydrochloride | 2 |
|  | Amoxapine | 1 |
|  | Clomipramine hydrochloride | 2 |
| **Benzodiazepine** |  |  |
|  | Bromazepam | 5 |
|  | Alprazolam | 4 |
|  | Ethyl loflazepate | 2 |
|  | Clotiazepam | 1 |
|  | Flunitrazepam | 1 |
|  | Nitrazepam | 1 |
|  | Diazepam | 1 |
|  | Lithium carbonate | 1 |
|  | Clonazepam | 1 |
| **DSS** |  |  |
|  | Aripiprazole | 6 |
| **DSA** |  |  |
|  | Blonanserin | 2 |
| **SDA** |  |  |
|  | Risperidone | 1 |
| **MARTA** |  |  |
|  | Quetiapine fumarate | 2 |
|  | Olanzapine | 1 |
| **Benzamide antipsychotics** |  |  |
|  | Sulpiride | 2 |
| **Branched fatty acid** |  |  |
|  | Sodium valproate | 2 |
| **Butyrophenone** |  |  |
|  | Pimozide | 1 |

Abbreviations: SSRI: Selective Serotonin Reuptake Inhibitor; NaSSA: Noradrenergic and Specific Serotonergic Antidepressant; DSS: Dopamine System Stabilizer; DSA: Dopamine Serotonin Antagonist; SDA: Serotonin-Dopamine Antagonist; MARTA: Multi-Acting Receptor Targeted Antipsychotics
